# Radiosynthesis, Stability, Lipophilicity, and Cellular Uptake Evaluations of [^131^I]Iodine-α-Mangostin for Breast Cancer Diagnosis and Therapy

**DOI:** 10.3390/ijms24108678

**Published:** 2023-05-12

**Authors:** Wiwit Nurhidayah, Eva Maria Widyasari, Isti Daruwati, Isa Mahendra, Toto Subroto, Nur Kusaira Khairul Ikram, Muchtaridi Muchtaridi

**Affiliations:** 1 Department of Chemistry, Faculty of Mathematics and Natural Sciences, Universitas Padjadjaran, Sumedang 45363, Indonesia; wiwit15001@mail.unpad.ac.id; 2 Research Collaboration Center for Theranostic Radiopharmaceuticals, Sumedang 45363, Indonesia; isti002@brin.go.id (I.D.); isam001@brin.go.id (I.M.); 3 Research Center for Radioisotope, Radiopharmaceutical, and Biodosimetry Technology, Research Organization for Nuclear Energy, National Research and Innovation Agency, South Tangerang 15310, Indonesia; evam001@brin.go.id; 4 Research Centre of Molecular Biotechnology and Bioinformatics, Universitas Padjadjaran, Sumedang 45363, Indonesia; t.subroto@unpad.ac.id; 5 Institute of Biological Sciences, Faculty of Science, Universiti Malaya, Kuala Lumpur 50603, Malaysia; nkusaira@um.edu.my; 6 Department of Pharmaceutical Analysis and Medicinal Chemistry, Faculty of Pharmacy, Universitas Padjadjaran, Sumedang 45363, Indonesia

**Keywords:** [^131^I]I-alpha mangostin, radiosynthesis, evaluations

## Abstract

The high rate of incidence and mortality caused by breast cancer encourage urgent research to immediately develop new diagnostic and therapeutic agents for breast cancer. Alpha mangostin (AM) is a natural compound reported to have anti-breast cancer properties. Its electron-donating groups structure allows it to be labeled with an iodine-131 radioisotope to develop a candidate of a diagnostic and therapeutic agent for breast cancer. This study aims to prepare the [^131I^]Iodine-α-mangostin ([^131^I]I-AM) and evaluate its stability, lipophilicity, and cellular uptake in breast cancer cell lines. The [^131^I]I-AM was prepared by direct radiosynthesis with Chloramine-T method in two conditions (A: AM dissolved in NaOH, B: AM dissolved in ethanol). Reaction time, pH, and mass of the oxidizing agent were optimized as crucial parameters that affected the radiosynthesis reaction. Further analysis was conducted using the radiosynthesis conditions with the highest radiochemical purity (RCP). Stability tests were carried out at three storage conditions, including −20, 2, and 25 °C. A cellular uptake study was performed in T47D (breast cancer cell line) and Vero cells (noncancerous cell line) at various incubation times. The results show that the RCP values of [^131^I]I-AM under conditions A and B were 90.63 ± 0.44 and 95.17 ± 0.80% (n = 3), respectively. In the stability test, [^131^I]I-AM has an RCP above 90% after three days of storage at −20 °C. A significant difference was obtained between [^131^I]I-AM uptake in T47D and Vero cells. Based on these results, [^131^I]I-AM has been prepared with high RCP, stable at −20 °C, and specifically uptaken by breast cancer cell lines. Biodistribution evaluations in animals are recommended as further research in developing [^131^I]I-AM as a diagnostic and therapeutic agent for breast cancer.

## 1. Introduction

In 2020, the GLOBOCAN database published by the International Organization for Research on Cancer (IARC) reported 2.3 million new cases and 685,000 deaths related to breast cancer. Breast cancer is the most observed cancer among women in 159 countries. It also caused the highest number of cancer deaths in 110 countries. The ratio of the incidence of breast cancer is one in four cancer cases among women, whereas the mortality is one in six for all cases of cancer death. These data lead to urgent research on the development of diagnostic and therapeutic agents for breast cancer [1,2].

A radiopharmaceutical is a drug formulated with radioactive compounds for diagnosis and therapy applications for various diseases, including breast cancer. As the name suggests, a radiopharmaceutical comprises a drug compound (pharmaceutical) and a radioactive compound (radioisotope) [3]. It can be used by oral, inhalation, or injection administration. Radioisotopes will emit radioactive rays for cancer diagnosis and therapy. The energy emitted can be detected using positron emission tomography (PET) or single-photon emission computed tomography (SPECT). Several radioisotopes emitting gamma rays, such as ^99m^Tc, ^111^In, ^18^F, and ^123^I, are used for diagnosis. Meanwhile, several radioisotopes, such as ^90^Y, ^211^At, ^177^Lu, and ^131^I, are used for therapy due to their emission of beta or alpha rays. Pharmaceutical compounds play a role in delivering radioisotopes to cancer cells due to their selective binding to a specific receptor in cancer cells [4,5,6,7]. Currently, many pharmaceutical compounds isolated from natural ingredients have been developed for the radiosynthesis of radiopharmaceuticals.

Natural compounds are known to have various secondary metabolites with anti-breast-cancer properties, such as terpenoids, flavonoids, alkaloids, phenolic compounds, nitrogen compounds, organic sulfur compounds, and xanthones [8]. Generally, they possess electron-donating groups, such as amines, phenols, and alkyl anilines, making them possible to be labeled with the iodine radioisotope through an electrophilic substitution by activating the carbon in the aromatic ring [9,10,11]. Several radiosyntheses of natural compounds with iodine radioisotope for breast cancer have been reported previously, including [^131^I]I-Lawsone from *Lawsonia inermis*, [^131^I]I-hydroxytyrosol from olive leaves extract, and [^131^I]I-Eugenol from *Syzygium aromaticum*. They showed a radiochemical purity (RCP) of greater than 90%. The cellular uptake values of [^131^I]I-Lawsone, [^131^I]I-hydroxytyrosol, and [^131^I]I-Eugenol on human breast cancer cells at 2 h incubation time were 5.23, 14.9, and 45.68%, respectively [12,13,14,15]. According to the data, the previous natural compounds are promising as diagnostic and therapeutic agents for breast cancer. To accelerate the advancement of radiopharmaceuticals research from natural compounds, exploration of other natural compounds that have potential as diagnostic and therapeutic agents for breast cancer is necessary to conduct.

Alpha mangostin (AM) is a xanthone derivative isolated from mangosteen rind (*Garcinia mangostana* L.), which is known to have antitumor activity in breast cancer. AM was reported to induce apoptosis in T47D cells by increasing the Bax/Bcl-2 ratio and increasing the expression of cleaved caspase-3 and caspase-9. In addition, AM can also inhibit PI3K/Akt and MAPK pathways by reducing the phosphorylation of ERa, HER2, c-Raf, PI3K, Akt, and ERK1/2 [16,17]. In vivo studies showed that AM can inhibit tumor growth in a BJMC3879luc2 cell BALB/c mice xenograft model at a 20 mg/kg/day concentration [18]. Furthermore, administration of 30 mg/kg/day AM orally reduced tumor volume by 74.1% in an LA7 cell Sprague Dawley rat xenograft model [19]. Due to its antitumor activity in breast cancer, AM can be labeled with a radioisotope as a diagnostic and therapeutic agent. AM will deliver radioactivity to the target cancer cells. Then, the radioisotope will play its role in the diagnosis and therapy of breast cancer.

**Figure 1:**
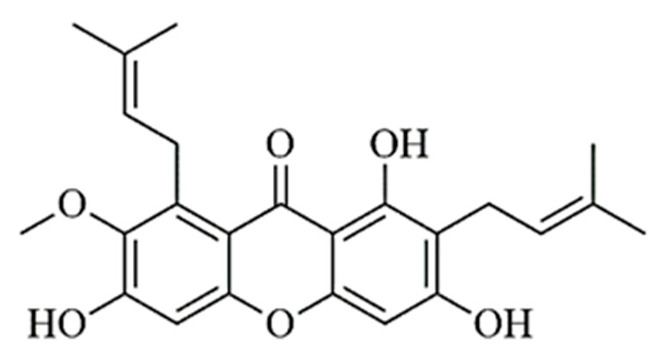
Structure of alpha mangostin (AM).

In this study, the radiosynthesis of [^131^I]I-Alpha mangostin ([^131^I]I-AM) was carried out by the direct method through an electrophilic substitution reaction. Iodine-131 is a radioisotope of iodine that emits beta 192 KeV and gamma 364 KeV with a half-life of 8.02 days. AM has electron-donating groups that allow iodine-131 to substitute the H atom at the ortho position of the hydroxyl group on the aromatic ring [20,21]. The AM structure is depicted in Figure 1. This study aimed to prepare the [^131^I]I-AM and evaluate its stability, lipophilicity, and cellular uptake in human breast cancer cell lines and normal cells (noncancerous cell lines). Further, [^131^I]I-AM is expected to have high radiochemical purity, stability, and uptake on human breast cancer cells to further develop it as a breast cancer diagnostic and therapeutic agent.

## 2. Results

### 2.1. Results of Radiosynthesis of [^131^I]I-AM

**Figure 2:**
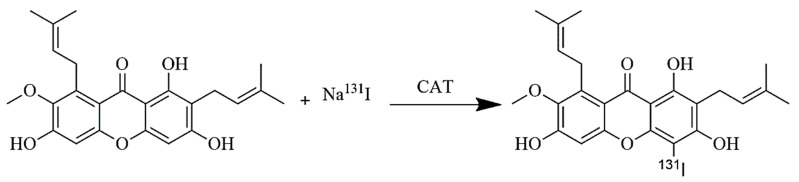
The prediction of radiosynthesis reaction of [^131^I] I-AM.

Radiosynthesis occurs through an electrophilic substitution reaction with chloramine-T as an oxidizing agent. The reaction is depicted in Figure 2.

### 2.2. Optimization of Parameters That Affected Radiosynthesis

#### 2.2.1. Optimization of pH

**Figure 3:**
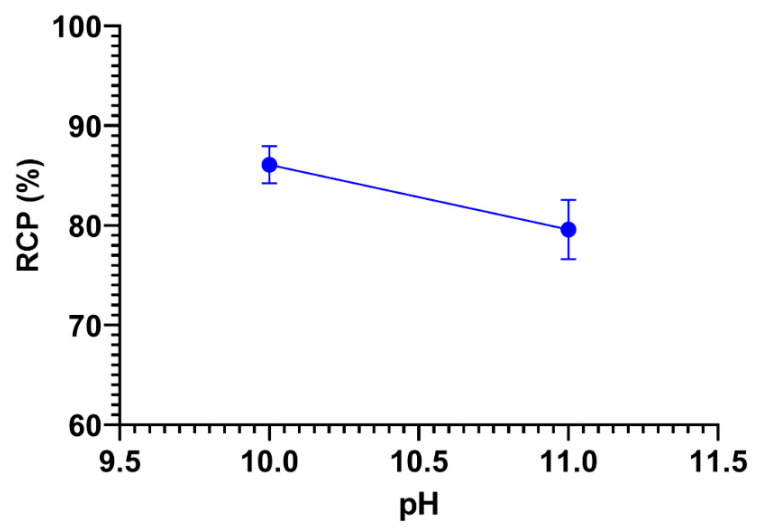
The effect of variations in pH on radiochemical purity of [^131^I]I-AM under condition A.

In condition A, AM was dissolved in NaOH so that the AM solution was in basic or alkaline condition. The pH was varied between pH 7, 8, 9, 10, and 11. However, the mixture precipitated at pH 7, 8, and 9, so only pH 10 and 11 were analyzed. The effect of reaction pH variations on radiochemical purity [^131^I]I-AM is shown in Figure 3. The radiochemical purity (RCP) values of pH 10 and 11 were 86.07 ± 1.87 and 79.58 ± 2.97%, respectively, whereas, in condition B, AM was dissolved in ethanol such that the reaction pH became 7 (neutral). Therefore, in condition B, pH optimization was not carried out.

#### 2.2.2. Optimization of Oxidizing Agent Mass

**Figure 4:**
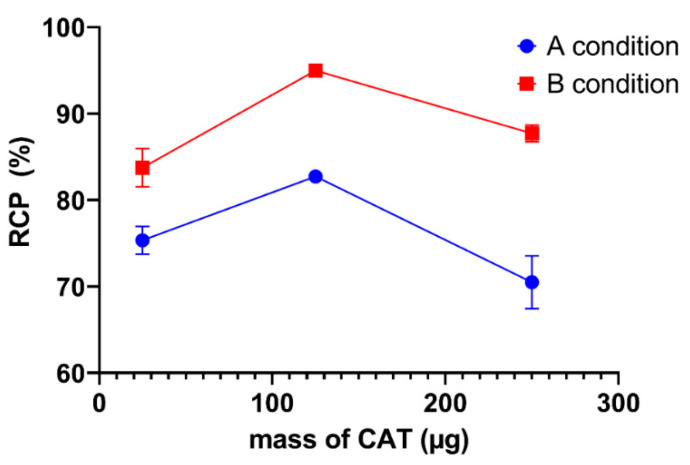
The effect of variations in CAT mass on radiochemical purity of [^131^I]I-AM under conditions A and B.

The mass of chloramine T was varied from 25 to 250 µg. The effect of variations in the mass of chloramine T on the radiochemical purity of ^131^I-AM under conditions A and B are shown in Figure 4. In condition A, the radiochemical purity at 25, 125, and 250 µg of CAT was 75.33 ± 1.59%, 82.72 ± 0.65, and 70.49 ± 3.07, respectively, whereas, in condition B, the RCP values obtained at 25, 125, and 250 µg of CAT were 83.73 ± 2.21, 94.98 ± 0.40, and 87.71 ± 0.99%, respectively. Both conditions showed the highest RCP at 125 µg of CAT.

#### 2.2.3. Optimization of Reaction Time

**Figure 5:**
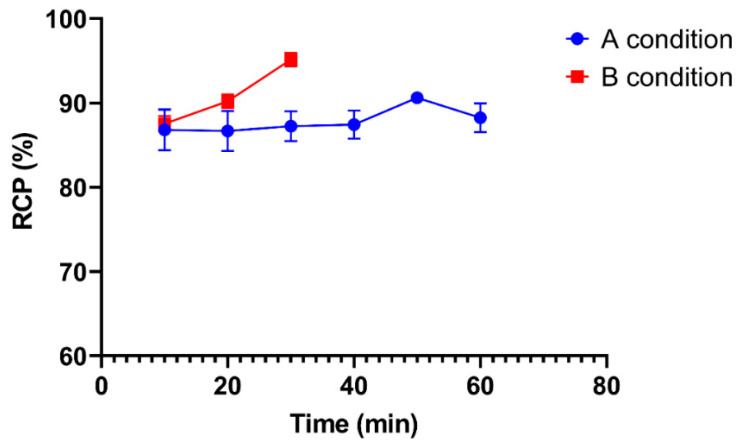
The effect of variations in reaction time on radiochemical purity of [^131^I]I-AM under conditions A and B.

The reaction time varied from 10 to 60 min. The effect of reaction time on RCP in conditions A and B is shown in Figure 5. In condition A, reaction times were varied to 10, 20, 30, 40, 50, and 60 min and the RCP values obtained were 86.82 ± 2.41, 86.69 ± 2.37, 87.25 ± 1.78, 87.45 ± 1.68, 90.63 ± 0.44, and 88.25 ± 1.70%, respectively. In condition B, the reaction time was varied to 10, 20, and 30 min and RCP was 87.54 ± 0.92, 90.20 ± 0.83, and 95.17 ± 0.80%, respectively. The RCP of [^131^I]I-AM under condition B was more than 95%, so longer incubation time variations were not required. The highest RCP values of [^131^I]I-AM under conditions A and B were obtained at 50 and 30 min of reaction time, respectively.

### 2.3. RCP Determination of [^131^I]I-AM

**Figure 6:**
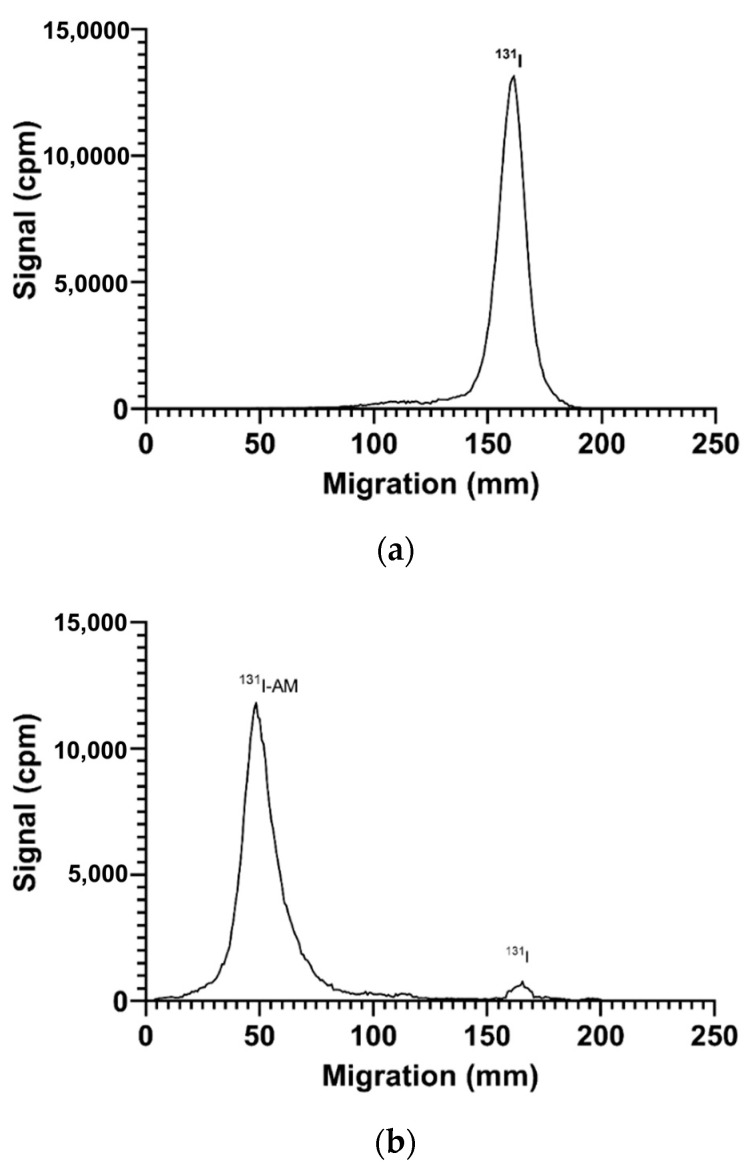
The migration of (**a**) iodine-131 and (**b**) [^131^I]I-AM at paper electrophoresis.

The determination of RCP by paper electrophoresis is shown in Figure 6. The migration of iodine-131 on paper electrophoresis is at 130–180 mm (Figure 6a), while the migration of [^131^I]I-AM is at 30–80 mm (Figure 6b). In Figure 6b, the radioactivity counted at 130–180 mm was assessed as a radiochemical impurity. Based on the analysis, the radiochemical purity obtained in conditions A and B was 90.63 ± 0.44 (n = 3) and 95.17 ± 0.80% (n = 3), respectively. The results of the optimization of [^131^I]I-AM radiosynthesis under conditions A and B are summarized in Table 1. Condition B produced a greater RCP than condition A. Therefore, condition B was used to prepare [^131^I]I-AM for further evaluation.

### 2.4. Stability Test of [^131^I]I-AM

**Figure 7:**
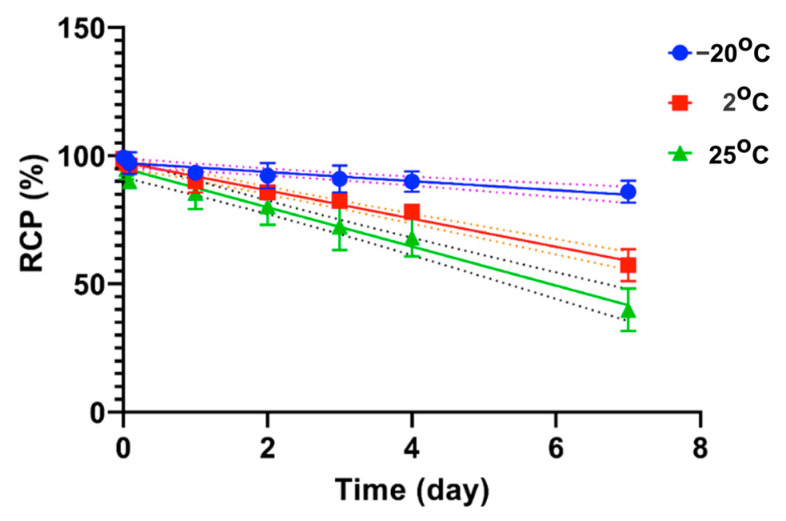
Stability of [^131^I]I-AM at −20 °C, 2 °C, and 25 °C.

Stability tests were conducted in the freezer (−20 °C), refrigerator (2 °C), and at room temperature (25 °C), as shown in Figure 7. At −20 °C, radiochemical purity was still above 90% after 3 days of storage, whereas, at 2 °C, the purity was still above 90% after 1 day of storage. In contrast, at 25 °C, the purity decreased to 85% after 1 day of storage.

### 2.5. Lipophilicity Evaluation

The log *p*-value of [^131^I]I-AM was determined to be 0.699 ± 0.19. The result showed that [^131^I]I-AM was a hydrophilic compound.

### 2.6. Cellular Uptake Evaluations

#### 2.6.1. Cellular Uptake Study on T47D Cell Line

**Figure 8:**
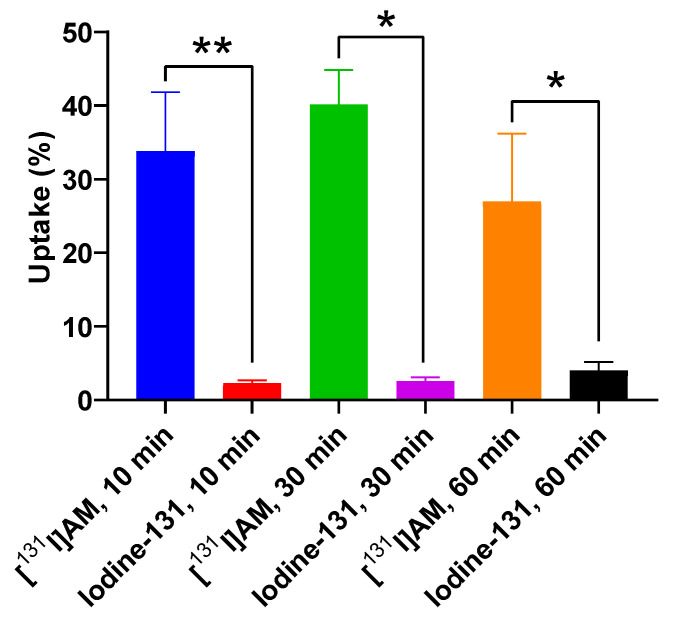
The cellular uptake of [^131^I]I-AM and iodine-131 on T47D cell line at 10, 30, and 60 min of incubation time. The statistical analysis of significance using *t*-test showed that the uptake of [^131^I]I-AM was significantly different from the uptake of iodine-131 on T47D cell line at the same time points (* showed significant difference with *p* < 0.05, and ** showed high significant difference with *p* < 0.01).

Cellular uptake was evaluated on T47D cells with 10, 30, and 60 min of incubation. As a comparison, the same test was carried out on iodine-131. The uptake of [^131^I]I-AM and iodine-131 on T47D cell lines are shown in Figure 8. The [^131^I]I-AM uptake at 10, 30, and 60 min of incubation was 33.80 ± 8.02, 40.12 ± 4.72, and 26.97 ± 9.23, respectively. Meanwhile, the iodine uptake at 10, 30, and 60 min of incubation was 2.3 ± 0.36, 2.56 ± 0.50, and 4.00 ± 1.18, respectively. According to the statistical analysis of significance using a *t*-test, the uptake of [^131^I]I-AM was significantly different from the uptake of iodine-131 at the same time points (*p* < 0.05).

#### 2.6.2. Cellular Uptake Study on Vero Cell Line (Noncancerous Cells)

**Figure 9:**
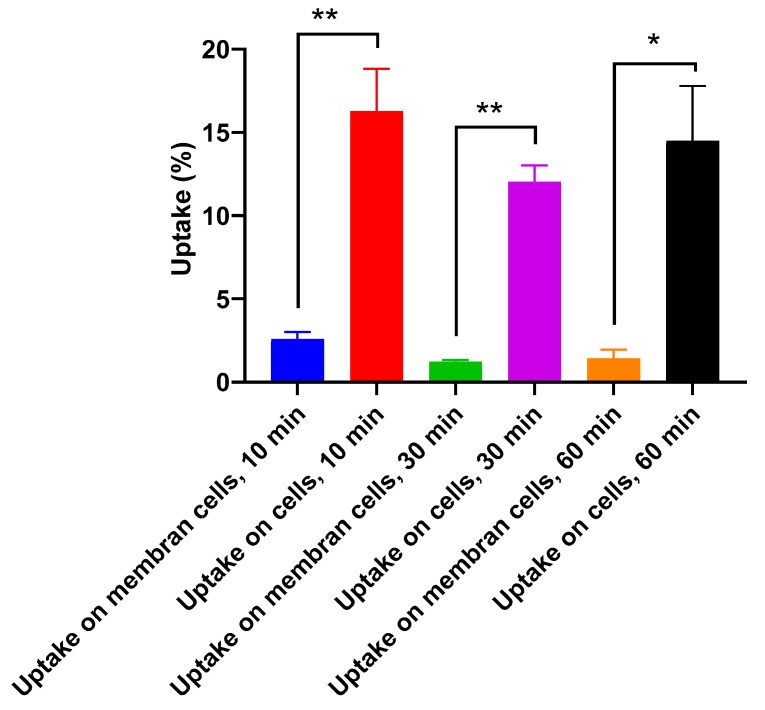
The cellular uptake of [^131^I]I-AM that was bound on the cell membrane and [^131^I]I-AM that was uptaken on Vero cell lines at 10, 30, and 60 min of incubation time (* showed significant difference with *p* < 0.05, and ** showed high significant difference with *p* < 0.01).

Cellular uptake was evaluated on Vero cells with 10, 30, and 60 min of incubation. The uptake of [^131^I]I-AM on Vero cell lines is shown in Figure 9. The [^131^I]I-AM that was bound on the cell membranes at 10, 30, and 60 min of incubation was obtained by the addition of glycine solution with percentages of 2.58 ± 0.42%, 1.23 ± 0.1%, and 1.43 ± 0.52%, respectively. Meanwhile, the [^131^I]I-AM uptake at 10, 30, and 60 min of incubation on Vero cells was 16.27 ± 2.55%, 12.03 ± 0.98%, and 14.50 ± 3.29%, respectively. According to the statistical analysis, the percentage of [^131^I]I-AM that was bound to the cell membrane was significantly different from the uptake [^131^I]I-AM on the Vero cells at the same time points (*p* < 0.05).

**Figure 10:**
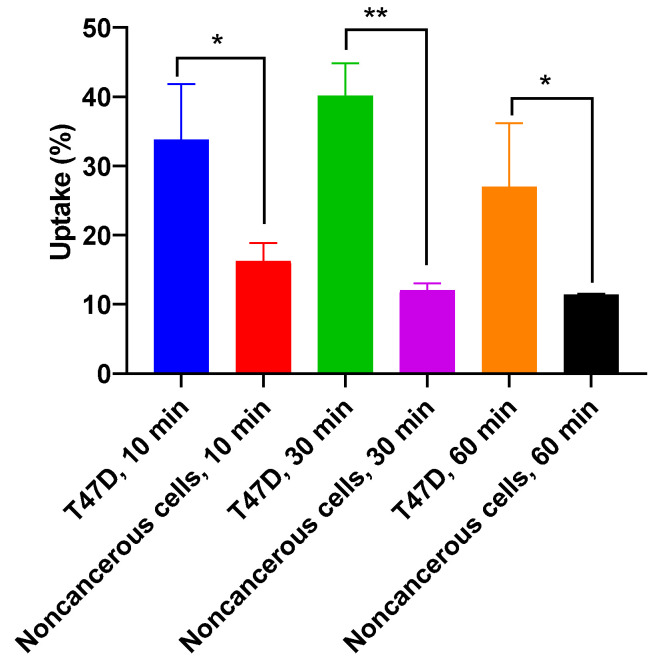
The cellular uptake of [^131^I]I-AM on T47D (breast cancer cell line) and Vero cells (noncancerous cell line) at 10, 30, and 60 min of incubation time. The uptake of [^131^]I-AM was significantly different from the uptake of iodine-131 at the same time points (* showed significant difference with *p* < 0.05, and ** showed high significant difference with *p* < 0.01).

The selectivity of [^131^I]I-AM against breast cancer cells was evaluated by comparing its uptake on T47D (breast cancer cell line) with Vero cells (noncancerous cells). Statistical analysis showed significant differences between the uptake of [^131^I]I-AM on T47D and the Vero cells at the same time points (*p* < 0.05) (Figure 10).

## 3. Discussion

This study aimed to prepare [^131^I]I-alpha mangostin ([^131^I]I-AM) and evaluate its stability and cellular uptake. AM is a natural compound that is known to possess breast cancer activity. Previously, the radiolabeling AM has also been carried out using a ^99m^Tc radioisotope with EDTA as a chelating agent. However, the radiolabeling results showed low radiochemical purity (RCP) (70.6 ± 2.87%) [22]. Radiolabeling AM with iodine-131 is expected to obtain a radiolabeled compound with high RCP.

AM has electron-donating groups that allow it to be labeled with an isotope of iodine by an electrophilic substitution mechanism. Usually, iodine is available as a NaI solution, so an oxidizing agent is needed to change the iodine in its electropositive form. One of the widely used oxidizing agents is CAT. Several critical parameters that affect reactions using CAT include pH, amount of CAT, and reaction time [23,24]. Selim et al. (2022) conducted radiosynthesis of [^131^I]I-shikonin using CAT as an oxidizing agent. The optimization of pH, amount of CAT, and reaction time contributed to the high RCP of the [^131^I]I-shikonin produced [25]. In order to obtain [^131^I]I-AM with a maximum RCP value, the pH, mass of CAT, and reaction time were optimized in this study.

AM is soluble in NaOH and ethanol. Therefore, radiosynthesis of [^131^I]I-AM was conducted in two conditions: AM dissolved in NaOH (condition A) and ethanol (condition B). The solvent used affects the pH of AM, so pH is the first parameter to be optimized. Radiosynthesis with the CAT is generally optimal under neutral or weakly acidic conditions. AM is already in a neutral pH in condition B, so pH optimization is not required. Under neutral or weakly acidic conditions, CAT will produce HOI, which reacts with NaI to form iodonium ion H_2_OI^+^, which will substitute for H on the ortho position of hydroxyl in the aromatic ring [24].

Meanwhile, in condition A, the AM was at pH 11, and the pH varied from 7–11 to obtain the optimum pH. The pH was adjusted with the addition of 0.1 N HCl. However, the precipitate was formed at pH 9 and below; therefore, pH variations could only be completed at pH 10 and 11. CAT will release hypochlorite ions in basic conditions, reacting with NaI to form HOI. HOI will assume a role in electrophilic substitution reactions [24]. The optimization results showed that the maximum RCP was obtained at pH 10 with a value of 86.07 ± 1.87%, and pH 11 produces a lower RCP because HOI is disproportionate in terms of forming iodate and iodide, which are unfavorable for radiosynthesis [24].

The following parameter that determines the success of the radiosynthesis reaction is the mass of CAT as an oxidizing agent. The radiosynthesis reaction requires a sufficient concentration of CAT to oxidize iodine. The critical concern is that CAT is a strong oxidizing agent. An excessive concentration of CAT may decrease radiochemical purity due to oxidative side reactions, such as polymerization, denaturation, and chlorination of pharmaceutical agents [24]. In the CAT mass variation, both conditions produced the maximum RCP value at 125 µg CAT mass. The maximum RCP values in conditions A and B were 82.72 ± 0.65 and 94.98 ± 0.40, respectively.

The others parameter is reaction time. Radiosynthesis is expected to occur quickly for the effectiveness of the radiolabeled compound preparation process. However, this time must be sufficient to produce the radiolabeled compounds with high radiochemical purity. In condition A, the time varies from 10 to 60 min. The optimum radiosynthesis conditions were obtained in 50 min with a radiochemical purity of 90.63 ± 0.44%. In condition B, the radiochemical purity obtained was above 95% at 30 min of incubation. Therefore, 30 min is considered to be the optimum time.

Radiochemical purity (RCP) was determined by paper electrophoresis. This method was chosen based on the difference in charge between the [^131^I]I-AM compound and the radiochemical impurities (free iodine). The results showed that free Iodine-131 had a negative charge, so it accumulated at the anode (the right peak), while [^131^I]I-AM was at the cathode (the left peak), as shown in Figure 6. After going through the optimization stages, condition B produces a higher RCP than condition A, with RCP values of 95.17 ± 0.80% and 90.63 ± 0.44 (n = 3), respectively. Condition B showed a higher RCP value than condition A in every stage of optimization for all parameters. One key point causing this is that the pH in condition B (with methanol solvent) is neutral, which is the optimum condition for radiosynthesis reactions using CAT as an oxidizing agent. Condition B in CAT optimization exhibited a higher RCP than condition A with the same amount of CAT. Optimization of reaction time also demonstrated that condition B requires a shorter time with a higher RCP than condition A. These parameters suggest that condition B is the most ideal condition for [^131^I]I-AM radiosynthesis. Therefore, condition B is used as the radiosynthesis condition for further evaluation.

The following evaluation is a stability test. This stability test was carried out by storing the [^131^I]I-AM compound in three conditions, including temperatures of −20, 2, and 25 °C. At certain time points, samples were taken, and their radiochemical purity was determined. The sample is defined to be stable if the RCP is greater than 90%. At −20 °C, the samples were stable after being stored for 3 days. Meanwhile, at 2 °C, the purity was still above 90 on the second day of storage. At 25 °C, the purity decreased after 3 h of storage. Based on the stability test, the stable storage condition for [^131^I]I-AM is −20 °C for 3 days.

The lipophilicity test showed that the log *p*-value of [^131^I]I-AM was 0.699 ± 0.19. It indicated that the compound tended to be hydrophilic. Lipophilicity is related to the prediction of absorption, distribution, metabolism, and elimination (ADME) parameters of radiopharmaceuticals before in vivo study. Arnott and S. L. Planey (2012) summarized the relationship between lipophilicity and these parameters. A pharmaceutical compound will have high water solubility if the log *p*-value is less than 3. Then, the compounds with log *p* −1 to 5.9 are predicted to have good permeability against cell membranes. Meanwhile, a log *p*-value of 0–3 indicates high bioavailability, and, to penetrate through the blood–brain barrier (BBB), they must have a log *p*-value of more than 2 [26]. Based on its log *p*-value, [^131^I]I-AM is expected to have high solubility, permeability, and bioavailability. In addition, a hydrophilic compound tends to have fast clearance through the kidney for its elimination [27].

The [^131^I]I-AM is intended for the treatment and diagnosis of breast cancer. Therefore, [^131^I]I-AM is required to be uptaken by breast cancer cells. Breast cancer is associated with several receptors, including hormone receptors (HRs) and human epidermal growth factor receptors 2 (HER-2). Hormone receptors consist of estrogen (ER) and progesterone (PR) [28]. Based on this, breast cancer is grouped into five groups: luminal A (HR+/HER2−), normal-like (HR+/HER2−), luminal B (HR+/HER2+), HER2 amplification and overexpression, and triple-negative or basal-likes (HR−/HER2−) [29]. T47D cells represent breast cancer in the luminal A group (ERα+, PR+/−, and HER2−). T47D cells are sensitive to the estrogen receptor alpha (ERα) in the cytosolic membrane. Therefore, T47D cells are widely used for in vitro tests and the induced xenograft cancer animal model [8].

As a radiopharmaceutical candidate, [^131^I]I-AM consists of AM as a pharmaceutical agent and iodine-131 as a radioactive agent. AM is required to have an affinity for breast cancer receptors so that it can be uptaken by cancer cells with overexpressed breast cancer receptors. A previous study by Kritsanawong et al. (2016) determined the cytotoxic activity of AM with the MTT assay on T47D cells. Cells were treated with AM concentrations of 7.5, 15, and 30 µM, with incubation times of 4, 8, 12, 16, 20, and 24 h. The results indicated an IC_50_ value of 7.5 M [16]. From cytotoxicity tests, AM can play a role as a pharmaceutical agent that delivers iodine-131 to target receptors since it has an affinity for breast cancer cell receptors. Iodine-131 will emit beta and gamma rays. Beta rays play a role in the therapeutic effect of radiopharmaceuticals. On the other hand, gamma rays contribute to diagnosis because they can be detected by PET and SPECT (in clinical applications) and can be counted by an automatic gamma counter in determining cellular uptake studies.

The [^131^I]I-AM cellular uptake test was performed on T47D cells at 10, 30, and 60 min of incubation. As a comparison, the same procedure was also carried out with iodine-131. The maximum uptake of [^131^I]I-AM was obtained in 30 min of incubation with an uptake percentage of 40.12 ± 4.72%. The uptake of [^131^I]I-AM at 10 and 60 min was 33.80 ± 8.02 and 26.97 ± 9.23, respectively. Meanwhile, the iodine-131 uptake at 10, 30, and 60 min of incubation was 2.3 ± 0.36, 2.56 ± 0.50, and 4.00 ± 1.18, respectively. The uptake of [^131^I]I-AM and iodine-131 at the same time points showed a significant difference in statistical analysis with a *p* < 0.05. This result shows that [^131^I]I-AM can be uptaken into T47D breast cancer cells.

Aside from T47D breast cancer cells, the cell uptake evaluation was also performed on Vero cells, a noncancerous cell line. In Vero cells, the highest uptake was obtained at 10 min of incubation with an uptake percentage of 16.27 ± 2.55%. Meanwhile, the uptake at 30 and 60 min of incubation was 12.03 ± 0.98% and 14.50 ± 3.29%, respectively. In addition, the amounts of [^131^I]I-AM bound to the cell membrane at 10, 30, and 60 min of incubation represented percentage values of 2.58 ± 0.42%, 1.23 ± 0.1%, and 1.43 ± 0.52%, respectively.

The [^131^I]I-AM is expected to be a therapeutic and diagnostic agent for breast cancer. Therefore, it must be uptaken specifically against breast cancer cells. A comparison of [^131^I]I-AM uptake in T47D breast cancer cells and the noncancerous Vero cell line was performed statistically to identify its specificity against breast cancer cells. The [^131^I]I-AM showed a higher percentage of uptake in T47D compared to Vero cells. The difference in uptake showed a significant difference with a *p* < 0.05. This result shows that it was uptaken specifically on T47D breast cancer cells. Breast cancer can be accurately diagnosed if [^131^I]I-AM has a selective uptake on cancer cells compared to normal cells. In addition, its specific uptake expected [^131^I]I-AM to be a safe radiopharmaceutical that does not damage normal cells during therapeutic applications. Thus. [^131^I]I-AM could provide a new potential diagnostic and therapeutic agent for breast cancer. The further research that is recommended in developing [^131^I]I-AM as a diagnostic and therapeutic agent for breast cancer is biodistribution evaluation in animals to determine [^131^I]I-AM in vivo stability and its accumulation in organs and tumor tissue.

## 4. Materials and Methods

### 4.1. Materials

AM was purchased from Biopurify (China), Na^131^I was irradiated by Serpong-Siwabessy Multi-Purpose Reactor and processed in the Research Center for Radioisotope and Radiopharmaceutical Technology laboratory, Nuclear Energy Research Organization, National Research and Innovation Agency, chloramine T was purchased from Sigma Aldrich (USA), and sodium metabisulfite, benzene, ethanol, chloroform, disodium hydrogen phosphate, sodium dihydrogen phosphate, universal pH paper, and sodium hydroxide were purchased from Merck (Germany).

### 4.2. Study Design

**Figure 11:**
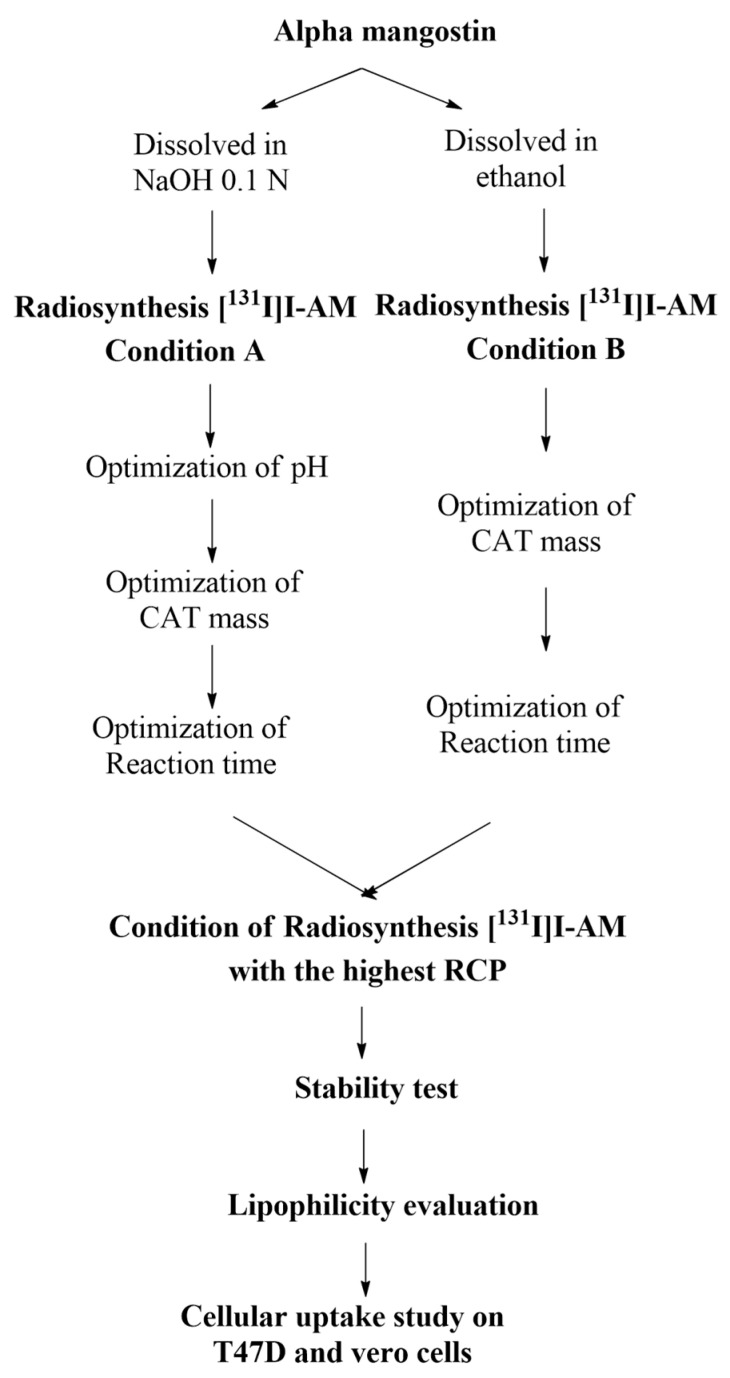
The flowchart of the study.

This study prepared [^131^I]I-AM by radiosynthesis using a direct method with Chloramine-T or CAT as an oxidizing agent based on our patent submission no. P00202010741. AM was dissolved in NaOH (condition A) and ethanol (condition B). AM in both conditions was labeled with iodine-131. The parameters that affected radiosynthesis, including pH, the mass of CAT as the oxidizing agent, and reaction time from each condition, were optimized to obtain optimum radiosynthesis conditions. Radiochemical purity (RCP) of [^131^I]I-AM was analyzed with paper electrophoresis. Condition with the highest RCP was used for the stability and cellular uptake on T47D cells evaluations. The stability test was carried out in three storage conditions within seven days. A cellular uptake study on T47D cell lines was conducted at incubation times of 10, 30, and 60 min. The flowchart of this study is illustrated in Figure 11.

### 4.3. Radiosynthesis of [^131^I]I-AM

Condition A: AM was dissolved in NaOH (0.65 mg/mL), and iodine-131 (3.7 mBq) was added. Chloramine T (2.5 mg/mL) was then added to the mixture and shaken for 10–60 min using Vortex. After that, Na_2_S_2_O_5_ (3.25 mg/mL) was added to stop the reaction. The RCP was obtained by electrophoresis.

Condition B: AM was dissolved in ethanol (0.65 mg/mL) and iodine-131 (3.7 mBq) was added. CAT (2.5 mg/mL) was then added to the mixture and shaken for 10–60 min using Vortex. After that, Na_2_S_2_O_5_ (3.25 mg/mL) was added to stop the reaction. The RCP was obtained by electrophoresis.

### 4.4. Optimization of Parameters That Affected Radiosynthesis

#### 4.4.1. Optimization of pH

The pH was optimized by varying the pH conditions from 7 to 11. The radiochemical purity of the product was analyzed with electrophoretic paper and counted by a TLC scanner. The pH of the reaction that produces the highest RCP value is determined as the optimum pH and used for further optimization.

#### 4.4.2. Optimization of Oxidizing Agent Mass

The oxidizing agent optimization was conducted by varying the CAT mass in the radiosynthesis to 25, 75, 100, and 125 µg. The radiochemical purity of the product was analyzed with electrophoretic paper and counted by a TLC scanner. The mass of CAT that produces the highest RCP value is determined as the optimum CAT mass and used for further optimization.

#### 4.4.3. Optimization of Reaction Time

The reaction time was optimized by varying the reaction time from 10 to 60 min. The radiochemical purity of the product was analyzed with electrophoretic paper and counted by a TLC scanner. The reaction time with the highest RCP value is determined as the optimum reaction time.

### 4.5. RCP Determination of [^131^I]I-AM

Radiochemical purity was determined by paper electrophoresis. The [^131^I]I-AM was spotted on Whatman 1 paper. The paper was set on an electrophoresis device with a stationary phase of phosphate buffer (0.25 M pH 7.4). The current was 10 A, the voltage was 10 volts for each paper, and the running time was 60 min. The paper was dried in the oven and analyzed with a TLC scanner. The following equation determined radiochemical purity:

Radiochemical impurity (%) = (Number of count impurity)/(Total number of count) × 100%

RCP (%) = 100 − radiochemical impurity (%)

### 4.6. Stability Test of [^131^I]I-AM

The stability test was carried out by storing [^131^I]I-AM at different temperatures, including a freezer (−20 °C), refrigerator (2 °C), and room temperature (25 °C). Samples were tested at 1–7 days, and radiochemical purity was determined by paper electrophoresis.

### 4.7. Lipophilicity Evaluation

Into centrifugation tube containing 2 mL of octanol and 2 mL of physiological NaCl 0.9% pH 7.4, [^131^I]I-AM was added. The mixture was shaken with a vortex stirrer for 1 min, then centrifuged at 3000 rpm for 10 min. A total of 50–100 µL of each fraction (octanol and NaCl) were taken and measured using a gamma counter. The octanol phase was transferred to another tube, and 0.9% physiological NaCl solution was added to equal volume. The experiment was repeated until a relatively constant value of the partition coefficient was obtained. A comparison of the radioactivity in the octanol phase and the NaCl phase shows the amount of lipophilicity, expressed as the partition coefficient of the preparation, which can be calculated by the following equation:

$$\mathrm{Partition}\;\mathrm{coefficient}=\frac{Radioactivity\;in\;octanol\;phase}{Radioactivity\;in\;NaCl\;phase}$$

### 4.8. Cellular Uptake Evaluations

#### 4.8.1. Cellular Uptake Study on T47D Cell Line

Cells preparation: Cell line T47D was obtained from the Laboratory of Cell Culture and Cytogenetics, Faculty of Medicine, Universitas Padjadjaran. T47D cells were prepared in RPMI medium 24 h before the experiment in a 24-well plate [30].

Experiment: Cell medium was removed using a micropipette, then rinsed with 1 mL Hanks′ Balanced Salt solution (HBSS) buffer. Further, 10µL of sample solution consisting of [^131^I]I-AM and standard ^131^I was added to the triple wells. Incubation at room temperature was carried out with selected time ranges of 10, 30, and 60 min, and 1 mL of HBSS was added, and the HBSS solution was discarded to remove any remaining radioactive compounds. Then, the cells were lysed by adding 60 µL of 0.2 N NaOH. Then, the radioactivity of each cell lysate was measured with an automatic gamma counter. Cell uptake from samples was calculated by the following equation:

$$\mathrm{Cell}\;\mathrm{uptake}\;(\%)=\frac{Radioactivity\;of\;cell\;lysate}{Radioactivity\;of\;standar}\;\times \;100\%$$

#### 4.8.2. Cellular Uptake Study on Vero cell Line (Noncancerous Cells)

Cells preparation: Cell line Vero was obtained from the Elabsciences Biotechnology Inc. Houston, TX, USA. Vero cells were prepared 24 h before the experiment in a 24-well plate.

Experiment: Cell medium was removed using a micropipette, then rinsed with 1 mL Hanks′ Balanced Salt solution (HBSS) buffer, and 10 µL of sample solution consisting of [^131^I]I-AM and standard ^131^I was added to the triple wells. Incubation at room temperature was carried out with selected time ranges of 10, 30, and 60 min, and 1 mL of HBSS was added, and the HBSS solution was discarded to remove any remaining radioactive compounds. Glycine was added and the solution was collected. After that, the cells were lysed by adding 60 µL of 0.2 N NaOH. Then, the radioactivity of each solution and cell lysate was measured with an automatic gamma counter. The percentage of [^131^I]I-AM bound to membrane cells was calculated by following equation:

$${[}^{131}I]I-\mathrm{AM}\;\mathrm{bound}\;\mathrm{to}\;\mathrm{membrane}\;\mathrm{cells}\;(\%)=\frac{Radioactivity\;of\;glycine\;solution}{Radioactivity\;of\;standard}\;\times \;100\%$$

Additionally, the percentage of cellular uptake of [^131^I]I-AM was determined by the following equation:

$$\mathrm{Cell}\;\mathrm{uptake}\;(\%)=\frac{Radioactivity\;of\;cell\;lysate}{Radioactivity\;of\;standar}\;\times \;100\%$$

#### 4.8.3. Statistical Analysis

All data are presented as the mean ± standard deviation (SD) of at least three independent measurements. Cellular uptake was analyzed using a *t*-test (GraphPad Prism, San Diego, CA, USA). Significance was assigned at *p* < 0.05.

## 5. Conclusions

The [^131^I]I-AM was prepared by radiosynthesis using direct method iodination with CAT as the oxidizing agent. Optimization of pH, the mass of CAT, and reaction time showed that radiosynthesis AM, which dissolved in ethanol (condition B), has a greater RCP compared to AM in NaOH (condition A), with RCP values of 95.17 ± 0.80% and 90.63 ± 0.44 (n = 3), respectively. In the stability test, [^131^I]I-AM was stable after three days of storage at −20 °C. A significant difference was obtained between [^131^I]I-AM uptake in T47D cells and Vero cells, which showed that [^131^I]I-AM was specifically uptaken by breast cancer cell lines. Biodistribution evaluations in animals are recommended as further research in developing [^131^I]I-AM as a diagnostic and therapeutic agent for breast cancer.

## Figures and Tables

**Table 1:** The summary of optimization parameters that affected radiosynthesis of [^131^I]I-AM.

Parameters	Condition A	Condition B
pH	10	7
Mass of CAT	125 µg	125 µg
Reaction time	50 min	30 min
RCP (n = 3)	90.63 ± 0.44%	95.17 ± 0.80%

## Data Availability

Not Applicable.
